# TMEM38B Gene Mutation Associated With Osteogenesis Imperfecta

**DOI:** 10.7759/cureus.69021

**Published:** 2024-09-09

**Authors:** Mrouge Sobaihi, Abdullah K Habiballah, Abdulrahman M Habib

**Affiliations:** 1 Department of Pediatric, King Faisal Specialist Hospital and Research Centre, Jeddah, SAU

**Keywords:** homozygous mutation in the tmem38b gene, oi osteogenesis imperfecta, osteogenesis imperfecta xiv, tmem38b mutation, tmem38b osteogenesis imperfecta

## Abstract

Osteogenesis imperfecta is a genetic disorder characterized by decreased bone density, bone deformities, and fractures. It results from mutations in different genes, including all steps of collagen 1 synthesis and modifications. In addition, the gene is involved in the homeostasis of intracellular calcium. TMEM38B is a gene involved in the formation of a cation channel responsible for calcium entry intracellularly. Mutations in this gene are associated with osteogenesis imperfecta. However, this mutation has not been frequently discussed in the literature. In our study, we report a case of TMEM38B-associated autosomal recessive osteogenesis imperfecta in a child of a consanguineous family presented with a history of multiple prenatal and postnatal fractures. No other associated complications are present in our case.

## Introduction

Osteogenesis imperfecta is a genetic disorder characterized by a group of skeletal and extra-skeletal manifestations that are caused as a result of defective collagen type I [1]. The skeletal manifestations include decreased bone density, pathological bone fractures, short stature, and progressive bone deformity. The extra-skeletal complications can affect the teeth, joints, sclera, and the ear, leading to dentinogenesis imperfecta, hyperlaxity of joints, blue sclera, and conductive hearing loss, respectively [2]. Collagen type one is a triple-helical interstitial matrix protein that is involved in the formation of many tissues like bones, teeth, and skin. It is formed by a union of two chains called pro-alpha 1 and pro-alpha two chains, encoded by COL1A1 and COL1A2 genes, respectively [3]. Several mutations have been associated with abnormal collagen transcription, translation, post-translational modifications, cross-linkage, mineralization, and bone formation [4]. The genes of Osteogenesis Imperfecta (OI) are inherited in an autosomal dominant mechanism in 90% of cases. However, some genes are inherited in an autosomal recessive manner [3,5]. Some important genes that are inherited in an autosomal recessive mode are FKBP10, CRTAP, P3H1, PPIB, SERPINF1, and BMPI [6]. A rare mutation in the transmembrane protein 38B (TMEM38B gene) has been reported in the development of OI in the literature [7]. The TMEM38B gene encodes for a transmembrane protein in the endoplasmic reticulum, which is one of the two trimeric intracellular cation channels that are responsible for calcium regulation intracellularly. A mutation in TMEM38B is associated with disturbances in the calcium deposition intracellularly and the development of calcium-related bone disorders like osteogenesis imperfecta [8]. Very few studies have reported a TMEM38B-related OI; most of them are reported in the Middle East. Shaheen et al. revealed TMEM38B mutations in three multiplex families with autosomal recessive OI among Saudi families [9]. In addition, Volodarsky et al. also reported a deletion in the TMEM38B gene associated with autosomal recessive OI among 10 individuals from three Israeli Bedouin unrelated consanguine families [10]. This paper discusses the non-classical presentations of TMEM38B-associated OI, like the absence of blue sclera or hearing impairment. In addition, it highlights the contribution of the TMEM38B mutation in the development of OI among the Arab population in Saudi Arabia.

## Case presentation

**Figure 1:**
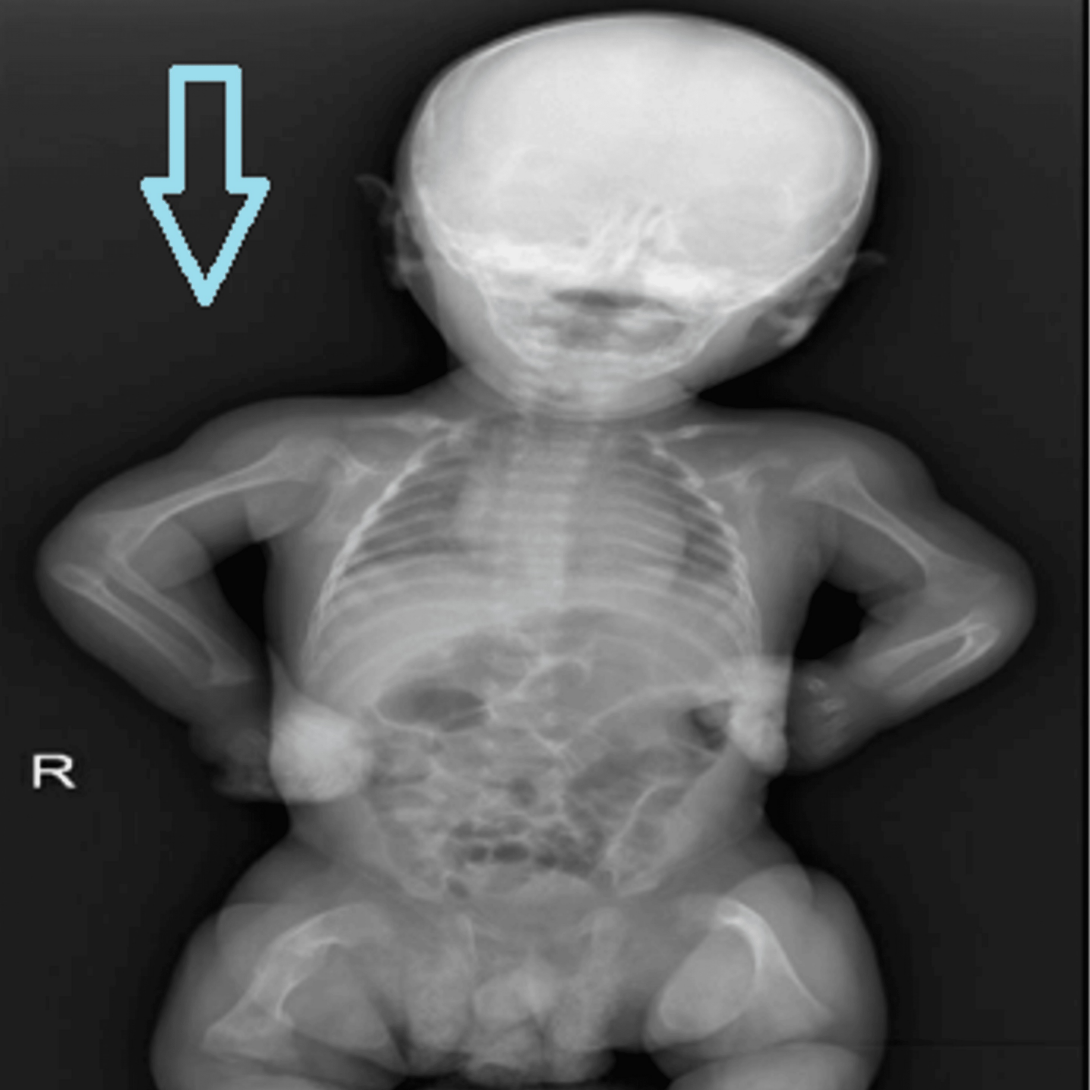
Showing diffuse severe osteopenia

**Figure 2:**
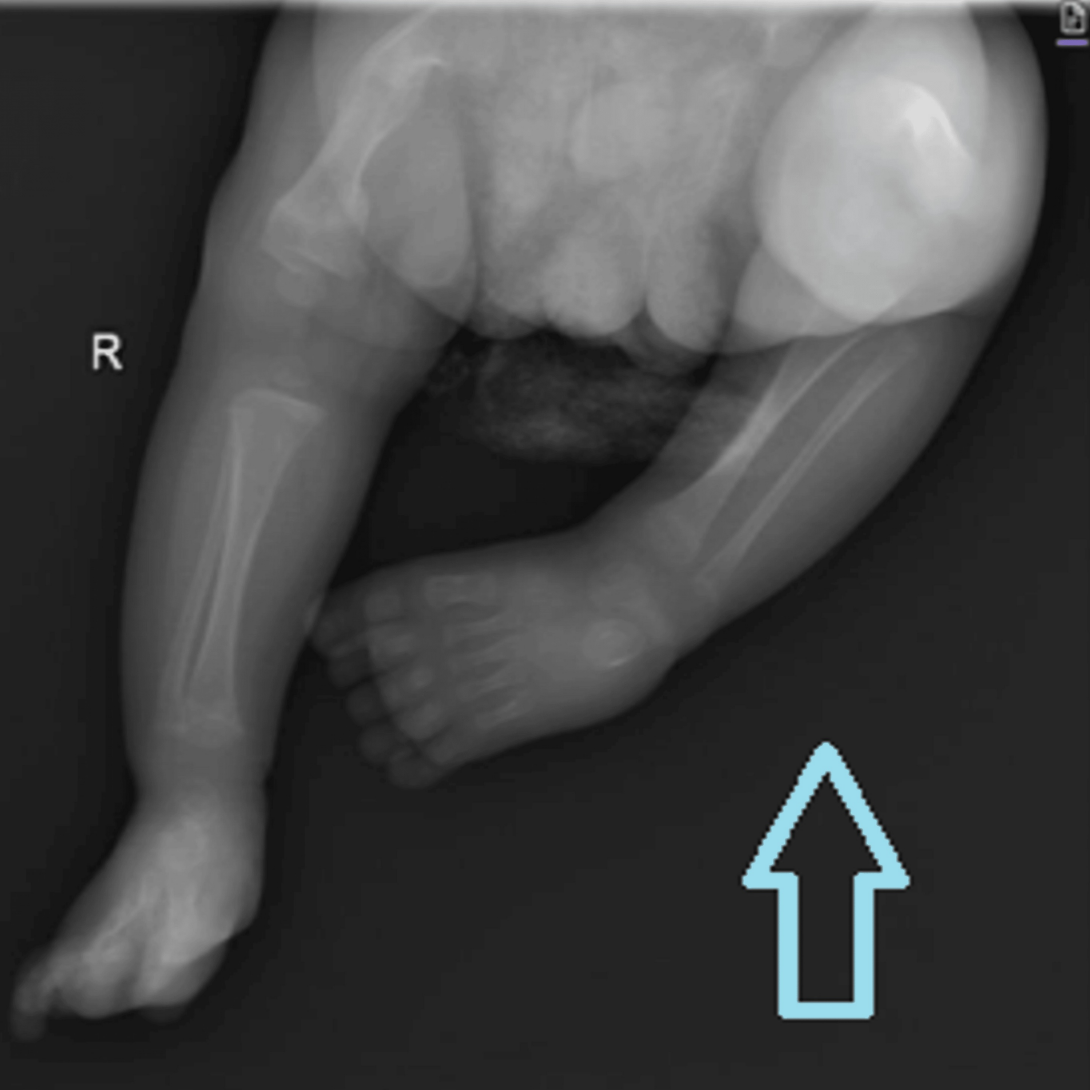
Showing diffuse severe osteopenia

A 17-month-old male patient presented to the endocrinology clinic at the age of one month with a history of multiple bone fractures for follow-up and further investigations. The boy was delivered by C-section due to an intrauterine fracture in the 8th month of pregnancy. He was delivered preterm in the 36th week of pregnancy with a birth weight of 2400 g and was admitted to the Neonatal Intensive Care Unit (NICU). His parents were a first-degree consanguineous couple with five healthy children. The mother had a history of one abortion and no history of stillbirth, and there were no reported cases of osteogenesis imperfecta or bone fractures in the family. His vital signs showed temperature, 37.1°C (axillary); pulse, 100 beats/min (brachial); blood pressure, 105/60 mmHg; and respiratory rate, 23 breaths/min. Physical examination revealed a normal-looking infant with no obvious abnormality, no chest wall deformity, and no blue sclera. His growth chart using the Saudi growth chart recorded a height of 47 cm (below the third percentile) and a weight of 3.6 kg (on the third percentile) in his first month, and a height of 76 cm (between the 10th and 25th percentile) and a weight of 9.4 kg (between the 10th and 25th percentile) at the age of 17 months. His first year of life growth velocity was 1.82 cm/month. An audiology screening showed normal hearing test results, and his skeletal survey revealed diffuse severe osteopenia (Figures 1, 2). The boy experienced his second fracture at the age of three weeks in the left femur.

Differential diagnosis

Several diseases can cause multiple fractures in infants. We tried to put the differential and exclude each one to get to the proper diagnosis. For example, preterm birth is characterized by decreased bone mineralization. However, the bone mineral density (BMD) usually returns to normal after one year of delivery, and this boy has decreased BMD after 16 months. Another differential is rickets. However, rickets have characteristic radiological findings, including cupping and fraying of the costochondral junctions and epiphyses, widening of the epiphysis, and cortical thinning, and none of these findings were found in the x-ray of the patient. Another differential could be osteomyelitis. However, osteomyelitis usually presents as a classical metaphyseal lesion in the long bones, and this patient reported no such findings in his x-ray. The final diagnosis that was considered is a genetic abnormality, especially with the positive family history of consanguinity and the history of intrauterine fractures in the 8th month of pregnancy. To ensure our diagnosis, we performed genetic testing to detect the affected genes [11].

Genetic testing

A written consent was obtained from the patient's family to perform a genomic sequencing. A peripheral blood sample was collected in an EDTA tube from the patient. A chromosomal microarray analysis was performed and revealed A homozygous intragenic deletion of TMEM38B (TMEM38B: NM_018112.3: exon 4 deletion: Chromosome 9: Position 108484750 - 108484908; Cytoband q31.2 - q31.2: Copy number 0: Length 159bp) was identified in this patient.

TMEM38B is a gene involved in the formation of a monovalent cation channel in the endoplasmic reticulum of the cell. It is a voltage-gated Na-K pump that is responsible for maintaining physiological levels of Na and K intracellularly. A defect in this channel is associated with disturbances in the level of Na and K intracellularly and Na-Ca exchange, subsequently. Mutations in the TMEM38B gene have been associated with disturbances in the level of intracellular calcium ions and the development of imperfect osteogenesis [12].

## Discussion

Osteogenesis imperfecta is a hereditary disorder associated with multiple gene mutations that may involve any step of collagen type 1 or affect calcium intracellular calcium homeostasis [6]. The first classification of OI was introduced by Sillence et al. when they classified OI into four main categories; class I OI is a dominantly inherited OI with blue sclera. It is clinically characterized by recurrent bone fractures and blue sclera; the second type is an autosomal recessive prenatal OI that is characterized by multiple intrauterine fractures, micromelia, and severe pulmonary disorders. Class III and IV are usually intermediate in severity. However, type III is more severe with multiple bone deformities and fractures [13]. A few years later, five additional types have been added based on the histological and molecular study of the genes involved in the pathogenesis of OI [14,15]. An alternative classification was developed based on the metabolic mechanism involved in the mutation, with five groups identified in this classification. Group A included all the mutations with defects in collagen synthesis or structure; Group B included the mutations with defects in collagen modification; Group C included the mutations associated with collagen folding and cross-linking defects; Group D compromised bone mineralization; and Group E included the defects in the development of osteoblasts and production of collagen [8,16]. Finally, the Online Mendelian Inheritance in Man (OMIM) Database developed a new classification based on all the previously mentioned classifications and identified different 22 subtypes of Osteogenesis Imperfecta [17].

Based on our genomic analysis and the OMIM classification, our patient has OI type XIV, because of a mutation in the TMEM38B gene that is responsible for forming a trimeric intracellular cation channel type B.

In our study, we found that the only clinical finding suggesting OI was the history of recurrent fractures and diffuse severe osteopenia with no impairment of the sclera or the hearing ability of the patient. These findings were consistent with Shaheen et al. and Ramzan et al., who reported no impairment of hearing or sclera among their cases [9,7]. The mutation has also been reported in a close geographical area where Volodarsky et al reported an autosomal recessive OI among three unrelated consanguineous families of the Israeli Bedouin, 4 of them had persistent grey-blue sclera and two had grey-blue sclera that disappeared with age. However, no impairment of hearing was reported among all of the children. [10] Rubinato et al. described a case report of an 11-year-old Albanian female presented with a clinical picture of osteogenesis imperfecta, including osteopenia, multiple bone fractures, and bone deformities. Genomic analysis revealed a deletion mutation in the TMEM38B gene. Clinical examination revealed mild conductive hearing loss and normal sclera. [18]. On the other hand, Fang Lv et al. reported the same mutation among three Chinese children from non-consanguineous families, presented with a clinical picture of recurrent fractures, decreased bone density, multiple deformities, growth retardation, and no signs of abnormal sclera or impaired hearing [19].

In our study, the absence of scleral affection and hearing impairment was consistent with the studies conducted among the Saudi population. However, different results were reported in other geographical areas. They also strengthen the hypothesis that TMEM38B mutations are associated with multiple bone deformities and autosomal recessive OI. However, further studies need to be conducted to confirm this association and calculate the estimated prevalence of TMEM38B mutations among Saudi citizens and their impact on the pathogenesis and subtypes of osteogenesis imperfecta.

## Conclusions

The TMEM38B mutation represents a rare variant of autosomal recessive osteogenesis imperfecta; it is commonly associated with multiple bone deformities, fractures, and growth retardation. TMEM38B mutation may be seen more frequently in consanguineous families given its mode of inheritance. Further research is needed to fully elucidate the prevalence and clinical features of this newly described form of autosomal recessive osteogenesis imperfecta. 

## References

[1] Forlino A, Cabral WA, Barnes AM, Marini JC (2011). New perspectives on osteogenesis imperfecta. Nat Rev Endocrinol.

[2] Cundy T (2012). Recent advances in osteogenesis imperfecta. Calcif Tissue Int.

[3] Marini JC, Blissett AR (2013). New genes in bone development: what's new in osteogenesis imperfecta. J Clin Endocrinol Metab.

[4] Rohrbach M, Giunta C (2012). Recessive osteogenesis imperfecta: clinical, radiological, and molecular findings. Am J Med Genet C Semin Med Genet.

[5] Van Dijk FS, Sillence DO (2014). Osteogenesis imperfecta: clinical diagnosis, nomenclature and severity assessment. Am J Med Genet A.

[6] Martínez-Glez V, Valencia M, Caparrós-Martín JA (2012). Identification of a mutation causing deficient BMP1/mTLD proteolytic activity in autosomal recessive osteogenesis imperfecta. Identification of a mutation causing deficient BMP1/mTLD proteolytic activity in autosomal recessive osteogenesis imperfecta.

[7] Ramzan K, Alotaibi M, Huma R, Afzal S (2021). Detection of a recurrent TMEM38B gene deletion associated with recessive osteogenesis imperfecta. Discoveries (Craiova).

[8] Marini JC, Reich A, Smith SM (2014). Osteogenesis imperfecta due to mutations in non-collagenous genes: lessons in the biology of bone formation. Curr Opin Pediatr.

[9] Shaheen R, Alazami AM, Alshammari MJ (2012). Study of autosomal recessive osteogenesis imperfecta in Arabia reveals a novel locus defined by TMEM38B mutation. J Med Genet.

[10] Volodarsky M, Markus B, Cohen I (2013). A deletion mutation in TMEM38B associated with autosomal recessive osteogenesis imperfecta. Hum Mutat.

[11] Jenny C (2006). Evaluating infants and young children with multiple fractures. Pediatrics.

[12] Cabral WA, Ishikawa M, Garten M (2016). Absence of the ER cation channel TMEM38B/TRIC-B disrupts intracellular calcium homeostasis and dysregulates collagen synthesis in recessive osteogenesis imperfecta. PLoS Genet.

[13] Sillence DO, Senn A, Danks DM (1979). Genetic heterogeneity in osteogenesis imperfecta. J Med Genet.

[14] Ward LM, Rauch F, Travers R (2002). Osteogenesis imperfecta type VII: An autosomal recessive form of brittle bone disease. Bone.

[15] Glorieux FH, Rauch F, Plotkin H (2000). Type V osteogenesis imperfecta: a new form of brittle bone disease. J Bone Miner Res.

[16] Forlino A, Marini JC (2016). Osteogenesis imperfecta. Lancet.

[17] Panzaru MC, Florea A, Caba L, Gorduza EV (2023). Classification of osteogenesis imperfecta: Importance for prophylaxis and genetic counseling. World J Clin Cases.

[18] Rubinato E, Morgan A, D'Eustacchio A, Pecile V, Gortani G, Gasparini P, Faletra F (2014). A novel deletion mutation involving TMEM38B in a patient with autosomal recessive osteogenesis imperfecta. Gene.

[19] Lv F, Xu XJ, Wang JY (2016). Two novel mutations in TMEM38B result in rare autosomal recessive osteogenesis imperfecta. J Hum Genet.

